# Antimicrobial, Antioxidant, Anti-Inflammatory, and Cytotoxic Activities of Propolis from the Stingless Bee *Tetragonisca fiebrigi* (Jataí)

**DOI:** 10.1155/2015/296186

**Published:** 2015-06-22

**Authors:** Jaqueline Ferreira Campos, Uilson Pereira dos Santos, Paola dos Santos da Rocha, Marcio José Damião, José Benedito Perrella Balestieri, Claudia Andrea Lima Cardoso, Edgar Julian Paredes-Gamero, Leticia Miranda Estevinho, Kely de Picoli Souza, Edson Lucas dos Santos

**Affiliations:** ^1^School of Environmental and Biological Science, Federal University of Grande Dourados, Rodovia Dourados-Itahum, Km 12, 79804-970 Dourados, MS, Brazil; ^2^Course of Chemistry, State University of Mato Grosso do Sul, Rodovia Dourados-Itahum, Km 12, 79804-970 Dourados, MS, Brazil; ^3^Department of Biochemistry, Federal University of São Paulo, Rua Pedro de Toledo 669, 04039-032 São Paulo, SP, Brazil; ^4^CIMO/ESA, Department of Biology and Biotechnology, Agricultural College of Bragança, Polytechnic Institute of Bragança, Campus Santa Apolónia, 5301-855 Bragança, Portugal

## Abstract

Propolis from stingless bees *Tetragonisca fiebrigi* found in Brazil is used in folk medicine by their nutritional and therapeutic properties. However, there are no scientific records evidencing such properties. The present study was designed to investigate the chemical composition and the biological properties of propolis from *T. fiebrigi*. For this, the chemical composition of the ethanol extract of propolis (EEP) was determined by GC-MS and presented phenolic compounds, alcohol, and terpenes as its major class compounds. The antimicrobial activity was accessed in gram-positive and gram-negative bacteria and in fungi, isolated from different biological fluids and reference strains. The EEP was active against all microorganisms and showed antioxidant activity by scavenging free radicals, inhibiting hemolysis and lipid peroxidation in human erythrocytes incubated with an oxidizing agent. The anti-inflammatory potential of the EEP was confirmed by inhibition of the hyaluronidase enzyme. The cytotoxic activity was concentration-dependent against K562 cells, with a predominance of death by necrosis. Taken together, these results show that propolis from *T. fiebrigi* has important therapeutic activities, which suggest its potential application in the pharmaceutical industry, as well as in health foods, beverages, and nutritional supplements.

## 1. Introduction

More than 200 species of stingless bees have been described in Brazil [1]. Among these species,* Tetragonisca fiebrigi*, popularly known in Brazil as* Jataí* or* abelha-ouro* (golden bee), covers a large part of the Brazilian territory, including the midwest, south, and southeast regions, as well as countries such as Argentina, Bolivia, and Paraguay [2, 3]. Recently, the taxonomic classification of this species has been revised. The species was previously described as* Tetragonisca angustula fiebrigi* subspecies along with* T. a. angustula*; however, molecular markers studies showed them to be distinct species [4, 5].

Among the species of stingless bees,* T. fiebrigi* is known as a good producer of honey and propolis, which are highly prized in folk medicine for their nutritional and therapeutic properties. Despite the popular use of these products, only the antibacterial and antioxidant properties of the honey produced by this species have been described in the literature [6, 7], and there are no scientific records of the therapeutic properties attributed to the propolis produced by these bees.

Propolis is a resinous substance produced by bees through the mixture of jaw secretions and the exudate collected from plant materials [8, 9]. This resin is used in the construction, maintenance, and asepsis of the nest [10]. There are records that ancient civilizations, such as the Incas, Greeks, Egyptians, and Romans, used propolis for its therapeutic properties, being described as antiseptic, healing, and antipyretic [11]. Additionally, this natural product has also been used in the preparation of drinks and foods for human nutrition, aiming at the improvement in health and disease prevention [12].

Indeed, recent studies expanded the knowledge on the therapeutic applications of propolis from different species of stingless bees, describing their antimicrobial [13, 14], antioxidant [15, 16], and antitumor [12, 17] properties.

These medicinal properties are directly related to the chemical composition. Studies of propolis from stingless bees describe the presence of phenolic acids, aromatic acids, terpenes, and carbohydrates [1, 16, 18]. The bee species and the botanical source from which the bees collect the resin are among the factors responsible for the physical, chemical, and biological characteristics of the propolis [12, 15].

Therefore, this study describes for the first time the chemical composition and antimicrobial, antioxidant, anti-inflammatory, and cytotoxic properties of propolis produced by the stingless bee* T. fiebrigi*.

## 2. Materials and Methods

### 2.1. Preparation of the Ethanol Extract of Propolis (EEP)

Propolis samples from* T. fiebrigi* bees (see Supplementary Material available online at http://dx.doi.org/10.1155/2015/296186) were collected from the state of Mato Grosso do Sul, in the midwest region of Brazil. Three colonies were identified, and six samples of propolis were collected with approximately 2.14 g each. The ethanol extract of propolis (EEP) was prepared with 4.5 mL of 80% ethanol per 1 g of propolis, kept in a water bath at 70°C in a sealed container until total dissolution, and subsequently filtered to obtain the EEP [19].

### 2.2. Chemical Analysis

One milligram of dry extract was reacted with 50 *μ*L pyridine + 100 *μ*L bis(trimethylsilyl) trifluoroacetamide (BSTFA) including 1% trimethylchlorosilane (TMCS) in a sealed glass tube for 30 min at 100°C to prepare samples for gas chromatography [20]. The procedure was performed in triplicate. Samples were injected and analyzed by gas chromatography-mass spectrometry (GC-MS). The procedure was performed in triplicate. The GC-MS analysis was performed on a gas chromatograph (GC-17A, Shimadzu, Kyoto, Japan) equipped with a mass spectrometer detector (QP 5050a), using DB-5 (J&W, 5% phenyl-dimethylpolysiloxane), fused-silica capillary column (30 m in length × 0.25 mm i.d., and 0.25 *μ*m film thickness), under the following conditions: carrier gas of helium (99.999% and flow rate 1.0 mL min^−1^), 1 *μ*L injection volume, split ratio (1 : 10), initial oven temperature set at 85°C, and heating from 85° to 315°C at 4°C min^−1^. The injector temperature was 280°C, and the quadrupole detector temperature was 315°C. The MS scan parameters included an electron-impact ionization voltage of 70 eV, mass range of 45–600* m/z*, and scan interval of 0.5 s. Temperature-programmed retention indices [21] were calculated using mixtures of linear alkane (C_8_–C_30_) as external references. The identifications were completed by comparing the mass spectra obtained in the QP5050 libraries. In some cases, when identified spectra were not found, only the structural type of the corresponding component was proposed on the basis of its mass-spectral fragmentation. When possible, reference compounds were cochromatographed to confirm GC-retention times.

### 2.3. Antimicrobial Activity

In the present study microorganisms isolated from biological fluids were used, collected in the Hospital Centre, and identified in the Microbiology Laboratory of Escola Superior Agraria de Braganca (ESA), but also reference strains were obtained from the authorised distributor of ATCC (LGC Standards S.L.U., Barcelona). The microorganisms were* Staphylococcus aureus* ATCC 43300,* S. aureus* ESA 654,* Staphylococcus epidermidis* ATCC 12228,* S. epidermidis* ESA 675,* Enterococcus faecalis* ATCC 43300,* E. faecalis* ESA 553,* Klebsiella pneumonia* ATCC 4352,* K. pneumoniae* ESA 154,* Pseudomonas aeruginosa* ATCC 15442,* P. aeruginosa* ESA 22,* Proteus mirabilis* ATCC 43300,* P. mirabilis* ESA 37,* Candida glabrata* ATCC 90030,* C. glabrata* ESA 123,* Candida albicans* ATCC 90028, and* C. albicans* ESA 345. The isolates were stored in Muller-Hinton medium plus 20% glycerol at −70°C, before experimental use. The inoculum for the assays was prepared by diluting cell mass in 0.85% NaCl solution, adjusted to 0.5 in MacFarland scale, confirmed by spectrophotometric reading at 580 nm for bacteria and 640 nm for yeasts. Cell suspensions were finally diluted to 10^4^ CFU/mL in order to use them in the activity assays. Antimicrobial tests were carried out as previously described by Silva et al. [22], using Nutrient Broth (NB) or Yeasts Peptone Dextrose (YPD) on microplate (96 wells). Propolis extracts were diluted in dimethyl sulfoxide (DMSO) and transferred into the first well and serial dilutions were performed. The inoculum was added to all wells and the plates were incubated at 37°C for 24 h (bacteria) and 25°C for 48 h (yeast). Amphotericin B and gentamicin were used as controls. In each experiment a positive control (inoculated medium) and a negative control (medium) and DMSO control (DMSO with inoculated medium) were introduced. Antimicrobial activity was detected by adding 20 *μ*L of 0.5% TTC solution (2,3,5-triphenyl-2H-tetrazolium chloride). The minimum inhibitory concentration (MIC) was defined as the lowest concentration of propolis extract that inhibited visible growth, as indicated by the TCC staining (dead cells are not stained by TTC).

In order to determine the Minimal Bactericidal Concentration (MBC) and Minimal Fungicidal Concentration (MFC), a portion of liquid (20 *μ*L) from each well in which colour changes were not verified was plated on NB or YPD and incubated at 37°C for 24 h (bacteria) and for 48 h (yeast). The lowest concentration that yielded no growth after this subculturing was taken as the MBC or MFC. All tests were performed in triplicate (*n* = 3) and results are expressed as mg/mL.

### 2.4. Antioxidant Activity

#### 2.4.1. ABTS Radical Scavenging Assay

Free radical scavenging capacity for EEP was studied as described by Re et al. [23], through the evaluation of the free radical scavenging effect on 2,2′-azinobis-(3-ethylbenzothiazoline-6-sulfonic acid) (ABTS) radical. The stock solutions included 7 mM ABTS solution and 140 mM potassium persulphate solution. The ABTS^∙+^ radical was then prepared by mixing the two stock solutions (5 mL of ABTS solution and 88 *μ*L potassium persulphate solution) and left for 12–16 h at room temperature in the dark. The solution was then diluted by mixing 1 mL ABTS^∙+^ radical with 50 mL ethanol absolute to obtain an absorbance of 0.70 nm ± 0.05 units at 734 nm using a spectrophotometer. Then, 20 *μ*L of EEP (0.1–500 *μ*g/mL) was mixed with 1980 *μ*L of the ABTS^∙+^ radical and the absorbance was taken at 734 nm after 6 min using a spectrophotometer. Ascorbic acid and butylated hydroxytoluene (BHT) were used as positive controls. Two independent experiments were performed in triplicate. The percentage of inhibition of the ABTS radical was calculated according to the following equation, where Abs_control_ is the absorbance of ABTS^∙+^ radical without the tested sample:

(1) % inhibition of ABTS=Abscontrol−AbssampleAbscontrol×100.

#### 2.4.2. Antioxidant Assay Using a Human Erythrocyte Model

*(1) Preparation of Erythrocyte Suspensions*. Following approval by the Research Ethics Committee (Comitê de Ética em Pesquisa; CEP) of the University Center of Grande Dourados (Centro Universitário da Grande Dourados; UNIGRAN), Brazil (CEP process number 123/12), 10 mL of peripheral blood was collected from healthy donors into sodium citrate-containing tubes and was subsequently centrifuged at 1500 rpm for 10 min. After centrifugation, the blood plasma and leukocyte layers were discarded, and the erythrocytes were washed 3 times with saline solution and centrifuged. Finally, 10% erythrocyte suspension was prepared in saline, to obtain a final 2.5% after the treatments.

*(2) Hemolytic Activity and Oxidative Hemolysis Inhibition*. The protective effect of the propolis extract was evaluated according to the method described by Campos et al. [16], with minor modifications. The assays were conducted with erythrocyte suspensions. The erythrocytes were preincubated at 37°C for 30 min in the presence of different concentrations of EEP or ascorbic acid (25–125 *μ*g/mL). Then, 50 mM 2,2′-azobis-(2-amidinopropane) dihydrochloride (AAPH) solution was added. A sample of 0.2% ethanol was used as a negative control. Total hemolysis was induced by incubating erythrocytes with distilled water. Basal hemolysis caused by EEP was assessed by incubating erythrocytes with the extract without the presence of AAPH, and the control was assessed in erythrocytes incubated only with 0.9% NaCl. This mixture was incubated at 37°C for 240 min, with periodical stirring. Hemolysis was determined after every 60 min of sample incubation for a total of 240 min; specifically, aliquots were removed, diluted in saline, and centrifuged at 1500 rpm for 10 min, after which the absorbance of the supernatant was read spectrophotometrically at 540 nm. The percentage hemolysis was measured with the formula *A*/*B* × 100, where (*A*) is the sample absorbance and (*B*) is the total hemolysis. Two independent experiments were performed in duplicate.

*(3) Inhibitory Efficiency against Lipid Peroxidation*. The protective effect of EEP against lipid peroxidation was evaluated according to the method described by Campos et al. [16], with minor modifications. Erythrocytes were preincubated at 37°C for 30 min with different concentrations of EEP or ascorbic acid (25–125 *μ*g/mL). A sample of 0.2% ethanol was used as a negative control. Next, 50 mM AAPH was added to the erythrocyte solution, which was then incubated at 37°C with periodical stirring. Dosage of malondialdehyde (MDA), a byproduct derived from lipid peroxidation, was determined after every 60 min of sample incubation for a total of 240 min. The samples were centrifuged at 1500 rpm for 10 min, and 500 *μ*L aliquots of the supernatant were transferred to tubes with 1 mL of 10 nmol thiobarbituric acid (TBA). As a standard control, 500 *μ*L of 20 mM MDA solution was added to 1 mL of TBA. The samples were incubated at 96°C for 45 min. The samples were then cooled, 4 mL of n-butyl alcohol was added, and the samples were centrifuged at 3000 rpm for 5 min. The sample supernatants were removed, and the absorbance was read at 532 nm. Two independent experiments were performed in duplicate. MDA levels in the samples were expressed in nmol/mL, obtained with the following formula:

(2) MDA=Absorbancesample×20×220.32AbsorbanceMDAstandard.

### 2.5. Anti-Inflammatory Activity: Hyaluronidase Assay

The inhibition of hyaluronidase activity was determined using the method described by Silva et al. [22]. The reaction mixture is constituted by 50 *μ*L of propolis' extract and 50 *μ*L (350 units) of hyaluronidase enzyme (type IV-S: bovine testes) was incubated at 37°C for 20 min. Then, calcium chloride was added (1.2 *μ*L, 2.5 × 10^−3^ M/L) to activate the enzyme and the mixture was incubated at 37°C for 20 min. To start the reaction 0.5 mL of hyaluronic acid sodium salt (0.1 M/L) was added. The mixture was incubated at 37°C for 40 min. After this, 0.1 mL of potassium tetraborate 0.8 M was added and it was incubated in water bath at ebullition for 3 min. The mixture was placed at 10°C and 3 mL of* p*-dimethylaminobenzaldehyde was added. Afterwards, it was incubated at 37°C for 20 min. Finally, the absorbance was measured at 585 nm using water as control. All the tests were performed in triplicate.

### 2.6. Cytotoxic Activity and Cell Death Profile

The K562 erythroleukemia cell line was grown in suspension in RPMI 1640 media (Cultilab, Campinas, Sao Paulo, Brazil) supplemented with 10% fetal bovine serum (FBS; Cultilab), 100 U/mL of penicillin, and 100 *μ*g/mL of streptomycin in a humidified atmosphere at 37°C in 5% CO_2_. The cytotoxic activity and cell death profile were evaluated according to the method described by Paredes-Gamero et al. [24], with minor modifications. Cells were seeded into 96-well plates (10^5^ cells/mL) and cultured in medium with 10% FBS in the absence or presence of EEP (31–500 *μ*g/mL) for 24 h. After this period, the K562 cells were washed with PBS and resuspended in annexin-labeling buffer (0.01 M HEPES, pH 7.4, 0.14 M NaCl, and 2.5 mM CaCl_2_). The suspensions were stained with annexin-FITC and propidium iodide (PI) (Becton Dickinson, Franklin Lakes, NJ, USA), according to the manufacturer's instructions. The cells were incubated at room temperature for 15 min. Ten thousand events were collected per sample, and the analyses were performed on a FACSCalibur flow cytometer (Becton Dickinson) with CellQuest software (Becton Dickinson). All the tests were performed in duplicate.

### 2.7. Statistical Analyses

The data are shown as the mean ± standard error of the mean (SEM) and were analyzed for statistical significant differences between the groups, using Student's *t*-test, comparing the treatment with the control, using the Prism 5 GraphPad Software. The results were considered significant when *P* < 0.05.

## 3. Results

### 3.1. Chemical Composition

**Figure 1:**
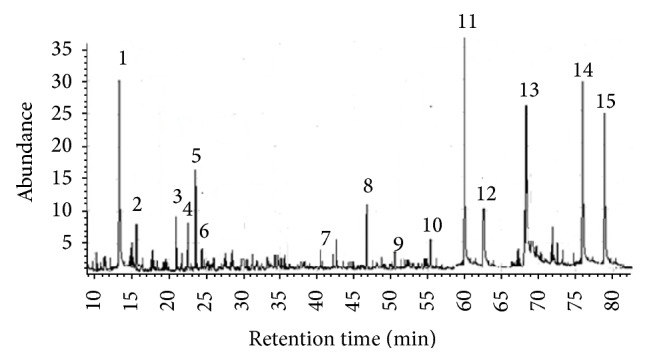
GC-MS profile of EEP from* T. fiebrigi*.

The identification of the propolis components was based on mass spectra interpretation, retention index, and basis data. The major compounds were benzoic and kaurenoic acids in relative area (Figure 1). The EEP components were identified and listed in Table 1.

### 3.2. Antimicrobial Activity

The EEP showed antimicrobial activity against all microorganisms evaluated (Table 2). The propolis from* T. fiebrigi* presented greater activity against the gram-positive bacteria than against gram-negative bacteria. The inhibition observed followed the sequence:* S. aureus* >* S. epidermidis* >* E. faecalis* >* P. mirabilis* >* K. pneumonia* >* P. aeruginosa*. Apart from this inhibitory activity, the EEP had bactericidal effects against all the organisms under study, ranging from 1.50 ± 0.14 mg/mL, for the reference strain of* S. aureus*, to 15.50 ± 0.29 mg/mL, for the isolated* P. aeruginosa*. Regarding the fungicidal activity, it was observed both in the reference stains and in those isolated from biological fluids.

### 3.3. ABTS Radical Scavenging Assay

Considering the presence of potentially antioxidant substances, an* in vitro* evaluation of the ABTS^∙+^ radical scavenging activity of EEP was performed at different concentrations. The 50% inhibitory concentration (IC_50_) and the maximal ABTS^∙+^ radical scavenging activity of EEP and the controls used are shown in Table 3. The IC_50_ of EEP was approximately five times higher than that of synthetic antioxidant BHT.

### 3.4. Oxidative Hemolysis Inhibition Assay

**Figure 2:**
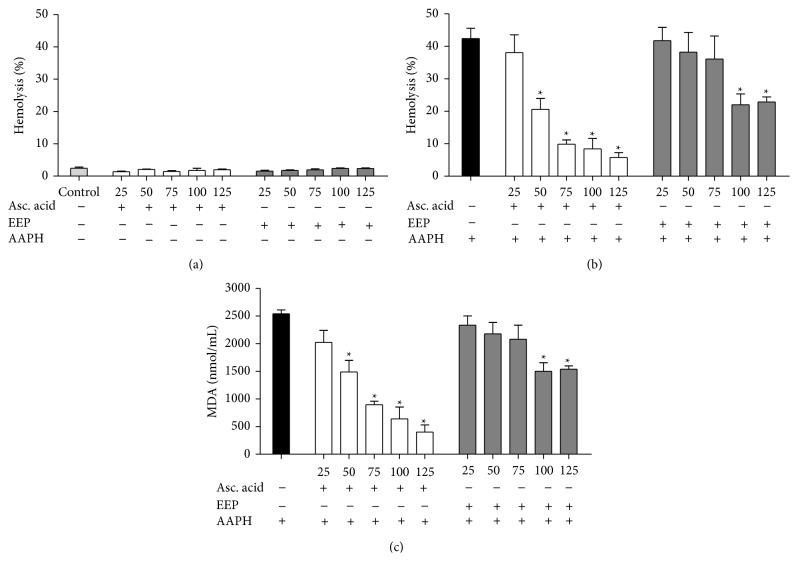
Human erythrocytes incubated at 240 min with ascorbic acid and EEP of* T. fiebrigi* (25–125 *μ*g/mL). (a) Hemolytic activity of treatments without the presence of AAPH and erythrocytes incubated only with 0.9% NaCl as control. (b) Hemolysis assessment after addition of AAPH in erythrocytes incubated with ascorbic acid and EEP. (c) Concentration of malondialdehyde (MDA) in nmol/mL after adding the oxidizing agent in erythrocytes incubated with different concentrations of treatments. ∗ represents statistically significant results (*P* < 0.05) when the treated group was compared to the AAPH group (erythrocytes incubated only with oxidizing agent).

The EEP was also evaluated for its hemolytic activity and ability to protect erythrocytes against AAPH-induced hemolysis. When erythrocytes were incubated only with ascorbic acid or EEP, no hemolysis was observed at the times and concentrations tested (Figure 2(a)), indicating that, at the concentrations evaluated, the extract is not toxic to this cellular model. Next, the ability to protect against AAPH-induced cell lysis of human erythrocytes was evaluated. The EEP showed antihemolytic activity at all experimental period in a concentration and time-dependent manner (data not shown). After a 240-min incubation of erythrocytes with EEP, a reduction of 46 ± 3.6% of hemolysis was observed in the highest concentration evaluated (Figure 2(b)).

### 3.5. Efficiency of EEP on the Inhibition of AAPH-Induced Lipid Peroxidation

The ability of the EEP to protect against AAPH-induced lipid peroxidation of human erythrocytes was evaluated through the dosage of MDA. The EEP showed reduced MDA levels at all experimental period in a concentration and time-dependent manner (data not shown). After a 240-min incubation of erythrocytes with EEP, a reduction of 39.5 ± 2.4% of MDA was observed in the highest concentration evaluated (Figure 2(c)).

### 3.6. Anti-Inflammatory Activity

**Figure 3:**
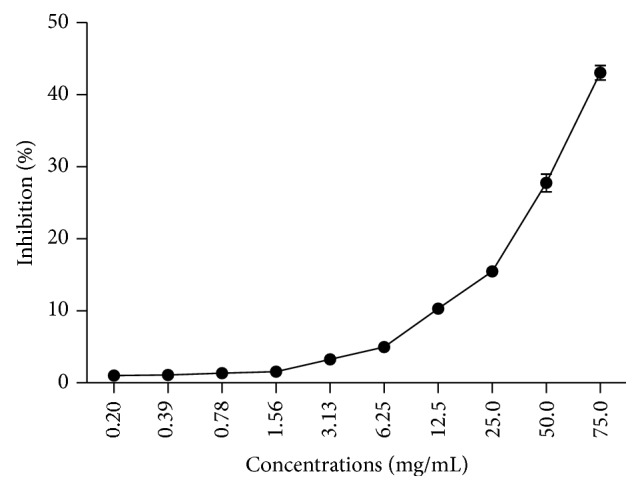
Anti-inflammatory activity for inhibition of the activity of hyaluronidase by the EEP in different concentrations.

It was observed that the EEP inhibited the hyaluronidase enzyme in a concentration-dependent manner. The highest concentration of extract evaluated (75 mg/mL) was able to inhibit 43.06 ± 3.06% of enzymatic activity (Figure 3).

### 3.7. Cytotoxic Activity and Cell Death Profile

**Figure 4:**
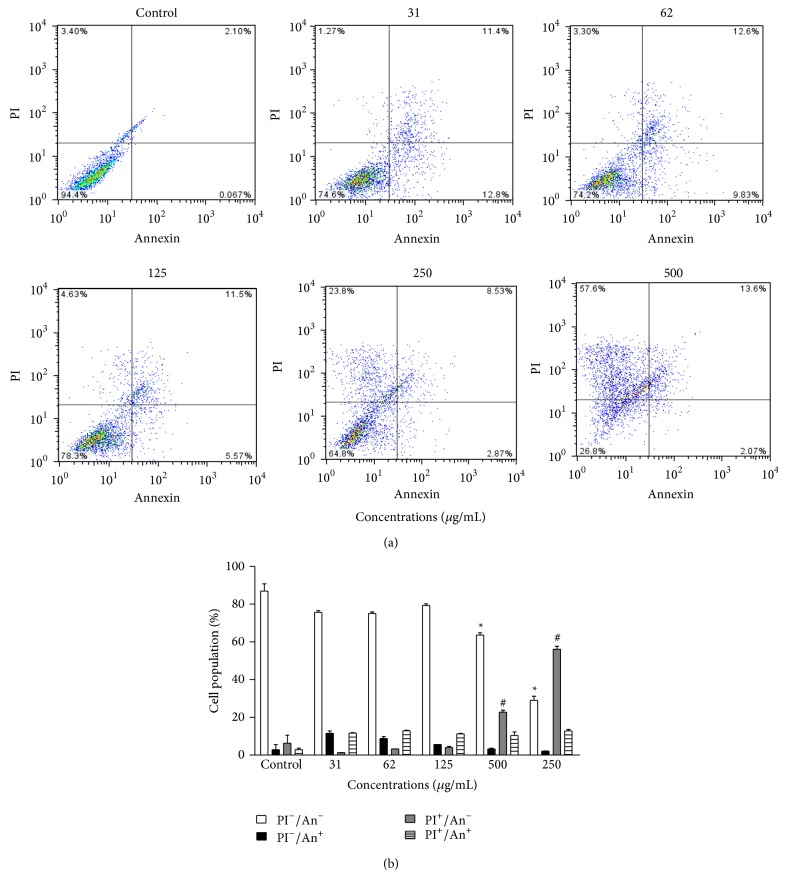
Cytotoxic action of EEP from* T. fiebrigi* against the K562 erythroleukemia cell line. (a) Representative diagrams obtained by flow cytometry of cells stained with annexin V-FITC/PI: the lower left quadrant (PI^−^/An^−^) represents the viable cells; the lower right quadrant (PI^−^/An^+^) represents the apoptotic cells; the upper left quadrant (PI^+^/An^−^) represents cells in necrosis; and the upper right quadrant (PI^+^/An^+^) represents cells in secondary necrosis. (b) Frequency of cell death, obtained from the corresponding diagrams of tested concentrations. ^*^ *P* < 0.05 treated group versus control viable cells. ^#^ *P* < 0.05 treated group versus control necrosis.

The K562 erythroleukemia cells treated with different concentrations of EEP were stained with annexin V-FITC/PI to verify the cell death profile. The EEP showed concentration-dependent cytotoxicity. At the highest concentration evaluated (500 *μ*g/mL), there was a reduction of 67 ± 2.5% of the viable cells (Figures 4(a) and 4(b)). The most effective cytotoxic concentrations of propolis, 250 and 500 *μ*g/mL, promoted cell death by necrosis (23 ± 1.0 and 56 ± 1.4%) and secondary necrosis (10 ± 1.8 and 13 ± 0.8%), respectively, indicating a concentration-dependent activity (Figures 4(a) and 4(b)).

## 4. Discussion

Propolis is a natural product that has a complex chemical composition. Substances in propolis composition are potentially responsible for its biological activity and may act synergistically and in an isolated manner. In this study, phenolic compounds, aromatic acids, alcohols, terpenes, and sugars were observed in the EEP from* T. fiebrigi*. These compounds have been identified in other studies of propolis of stingless bees found in Brazil [18, 25].

Even though the antimicrobial drugs have long been used either for prophylactic or therapeutic purposes, the emergence of drug-resistant microorganisms appears as a serious global concern. In this context, it is important to find other substances effective against pathogens resistant to the conventional therapies. Natural products, such as propolis, appear as a viable alternative, either by taking advantage of the synergistic effects of all the compounds present or by investing in specific isolated compounds.

Indeed, different studies have shown a positive correlation between phenolic compounds and terpenes with antimicrobial action [26, 27].

Phenolic compounds cinnamic acid,* p*-coumaric acid, and the diterpene kaurenoic acid identified in EEP from* T. fiebrigi* already were described as being responsible for the antibacterial activity of propolis from* Melipona quadrifasciata anthidioides* [26],* Tetragonisca angustula*, and* Apis mellifera* [18], other species of bees found in Brazil.

The phenolic compounds are described as important antibacterial agents that inhibit bacteria by promoting damage to the cell membrane and by inhibiting the synthesis of nucleic acids and the energy metabolism of these microorganisms. In addition, these compounds interfere with virulence factors of various bacteria, including enzymes, toxins, and other signaling molecules [28].

The EEP from* T. fiebrigi *showed antimicrobial activity against the gram-positive bacteria, gram-negative bacteria, and fungi, which is important for health maintenance, since that these microorganisms are responsible for common nosocomial infections in urinary tract, respiratory pneumonia, surgical site wound, gastrointestinal, and skin infections [22]. Propolis acts by inhibiting the growth and proliferation of bacteria [29]; however, gram-positive bacteria are more sensitive to the action of propolis than gram-negative bacteria, which may be due to structural differences of the cell wall [14, 25]. Mirzoeva et al. [30] suggested that the antimicrobial activity of propolis is species-dependent and that its constituents are able to promote an increase in membrane permeability and inhibit bacterial motility.

Among the antimicrobial activities of propolis, the EEP showed fungicidal properties against* C. glabrata* and* C. albicans*. Kujumgiev et al. [31] correlated the antifungal effect of Brazilian propolis with the presence of diterpene acids, triterpenes, aromatic acids, and phenolic compounds, which were also identified in propolis from* T. fiebrigi*.

Gallucci et al. [32] showed that many phenolic compounds inhibit fungal growth by interacting with the plasma or mitochondrial membrane of the fungus. de Castro et al. [33] reported that the mechanisms by which propolis promotes the death of fungi include vacuolar acidification and changes in the mitochondrial electron-transport chain, resulting in death by apoptosis; in turn, prolonged exposure results in secondary necrosis.

Recently, de Castro et al. [34] reported that propolis inhibits the morphological transition from yeast to hyphae, thus promoting the control of cell growth and differentiation, and induces cell death through the metacaspase pathway. Ramsdale [35] noted that many antifungal compounds promote cell death mediated by the apoptotic pathway and can be metacaspase dependent or independent. In addition, antifungal compounds stimulate the production of reactive oxygen species or interfere with calcium/calmodulin/calcineurin signaling. The discovery of new antifungal agents, especially those that promote cell death by different pathways, from natural sources is of great importance.

In addition to the antimicrobial activity, the EEP has antioxidant activity by the free radicals scavenging and the ability to inhibit the lysis of erythrocytes incubated with oxidizing agent, which was confirmed by lower generation of MDA, one of biochemical markers of lipid peroxidation.

The antioxidant properties presented by* T. fiebrigi* propolis extract are probably related to its chemical composition, especially by the presence of phenolic compounds as benzoic acid, cinnamic acid, and* p*-coumaric acid already described in the literature by capacity to capture free radicals [36, 37]. Other studies have shown that these compounds are responsible for the antioxidant activity by plant extracts [38–40] and propolis [41, 42]. According to Valente et al. [43], polyphenols from propolis are able to capture peroxyl radicals generated by AAPH oxidizing agent and inhibiting the lipid peroxidation process resulting in protection against oxidation of biomolecules present in the membrane of erythrocytes.

The reactive species of oxygen and nitrogen, when present in high levels in the body, are responsible for oxidative stress state, resulting in damage to cells and tissues [44]. Among the possibilities to neutralize the excess of these free radicals is consumption of natural foods with antioxidant properties, which are important auxiliary in the prevention of diseases related to oxidative stress, such as cancer, diabetes, and diseases associated with inflammation, such as rheumatoid arthritis and atherosclerosis [44, 45].

The propolis has also been used in the folk medicine in the prevention of inflammatory diseases, despite the lack of knowledge regarding the components that exhibit this activity. Inflammation is a biological response activated to restore tissue injury, such as pathogens, damaged cells, irritants, and free radicals. Propolis has been reported to possess anti-inflammatory activity both* in vitro* and* in vivo* by modulating key inflammatory mediators, inhibiting the production of proinflammatory cytokines, increasing anti-inflammatory cytokines, and blocking the activation of nuclear factor- (NF-) *κ*B [46, 47]. Activity was also observed in the propolis from* T. fiebrigi* by determination of the hyaluronidase enzyme, an indirect way to assess the anti-inflammatory activity. The hyaluronic acid is an important component of articular cartilage and plays an important role in tissues' renovation [22]. The degradation of hyaluronic acid by hyaluronidase enzyme may cause bone loss, inflammation, and pain [48].

The cytotoxic activity shown by EEP from* T. fiebrigi* can be related to the presence of phenolic compounds as coumaric acid, cinnamic acid, and derivatives. These compounds have already been described in propolis samples by their cytotoxic action against K562 (leukemia), HeLa (human cervical adenocarcinoma), and LNCaP (human prostate cancer) cell lines [49–51].

By investigating the cytotoxic potential of propolis from* T. fiebrigi* in K562 erythroleukemia cells, we observed the predominance of death by necrosis. This type of cell death can be promoted by opening mitochondrial permeability transition pores, leading to the depolarization and consequent cell death due to lack of ATP [52]. For many years, necrosis was seen as a kind of uncontrolled accidental death; however, necrosis is currently described as a programmed death that may contribute to the control of cancer-cell proliferation [53], in particular in cell lines resistant to conventional chemotherapy or cells showing resistance to apoptosis.

The K562 erythroleukemia cell line is described as exhibiting resistance to death by apoptosis [54]; therefore, alternative forms of cell death, such as necrosis, are necessary to inhibit the proliferation of these tumor cells [55]. Necrosis is caused by several biochemical and molecular signaling mechanisms, such as increases in levels of calcium in cell cytoplasm, increased interaction with serine-threonine kinase receptors, and increased abundance of reactive oxygen species, resulting in the disruption of organelles and cell death [56].

## 5. Conclusions

These results show that propolis from the stingless bee* T. fiebrigi* has important biological activities, including antimicrobial, antioxidant, anti-inflammatory, and cytotoxic activity against human erythroleukemia cell, suggesting its potential application in the pharmaceutical industry, as well as in health foods, beverages, and nutritional supplements.

## Supplementary Material

Graphical abstract: Presentation of the results more important of propolis from the *T. fiebrigi*.

## Figures and Tables

**Table 1:** Compounds identified in EEP from *T. fiebrigi *by GC-MS.

Peak	Retention time (min)	Molecular mass	Compound	% TIC^*∗*^
1	13.10	122	Benzoic acid	9.2
2	15.31	286	Cinnamyl caffeate	1.5
3	21.04	270	Benzyl caffeate	1.5
4	23.76	148	Cinnamic acid	3.6
5	23.88	150	Hydrocinnamic acid	1.3
6	24.51	178	Hydrocinnamic acid ethyl ester	0.6
7	40.86	164	p-Coumaric acid	0.4
8	47.03	232	3-Phenyl-p-coumaric acid	2.8
9	50.67	180	Fructose	0.6
10	56.12	180	Glucose	1.5
11	59.94	302	Kaurenoic acid	11.8
12	63.25	152	4-Methoxybenzoic acid	3.0
13	68.59	286	Retinol	8.1
14	76.89	386	Cholesterol	12.4
15	79.39	430	Tocopherol	7.4

^*∗*^TIC: total ion current. The ion current generated depends on the characteristics of the compounds.

**Table 2:** Minimum inhibitory concentration (MIC), minimum bactericidal concentration (MBC), and minimum fungicidal concentration (MFC) for same microorganisms gram-positive bacteria, gram-negative bacteria, and the fungi.

	EEP (mg/mL)		Gentamicin (*µ*g/mL)
	MIC	MBC	MBC
Gram-positive bacteria			
*Staphylococcus aureus *ATCC 43300	0.55 ± 0.05	1.50 ± 0.14	1.67 ± 0.17
*Staphylococcus aureus *ESA 654	0.65 ± 0.03	2.00 ± 0.14	1.33 ± 0.08
*Staphylococcus epidermidis* ATCC 12228	0.77 ± 0.02	1.75 ± 0.14	2.17 ± 0.17
*Staphylococcus epidermidis *ESA 675	0.88 ± 0.04	1.92 ± 0.36	2.00 ± 0.14
*Enterococcus faecalis *ATCC 43300	0.88 ± 0.07	3.8 ± 0.17	3.31 ± 0.20
*Enterococcus faecalis *ESA 553	1.02 ± 0.12	5.00 ± 0.14	3.33 ± 0.17
Gram-negative bacteria			
*Klebsiella pneumoniae *ATCC 4352	3.33 ± 0.22	11.58 ± 0.22	5.00 ± 0.29
*Klebsiella pneumoniae *ESA 154	3.75 ± 0.14	13.08 ± 0.22	5.08 ± 0.17
*Pseudomonas aeruginosa *ATCC 15442	5.83 ± 0.46	14.42 ± 0.22	4.91 ± 0.08
*Pseudomonas aeruginosa *ESA 22	7.91 ± 0.22	15.50 ± 0.29	5.00 ± 0.29
*Proteus mirabilis *ATCC 43300	2.25 ± 0.14	10.75 ± 0.38	8.67 ± 0.17
*Proteus mirabilis *ESA 37	3.08 ± 0.22	12.02 ± 0.30	8.92 ± 0.22

	EEP (mg/mL)		Amphotericin B (*µ*g/mL)
	MIC	MFC	MFC

Fungi			
*Candida glabrata *ATCC 90030	7.00 ± 0.29	9.00 ± 0.29	0.86 ± 0.08
*Candida glabrata *ESA 123	7.91 ± 0.27	11.83 ± 0.40	1.37 ± 0.19
*Candida albicans *ATCC 90028	7.90 ± 0.31	8.00 ± 0.29	0.75 ± 0.03
*Candida albicans *ESA 345	9.25 ± 0.38	12.91 ± 0.22	0.75 ± 0.03

Values are shown as means ± SEM (*n* = 3).

**Table 3:** IC_50_ and maximum activity of ABTS radical scavenging of standard antioxidants and of EEP.

Sample	IC_50_ (*µ*g/mL)	Maximum inhibition	
		%	*µ*g/mL
Ascorbic acid	1.3 ± 0.2	99.5 ± 0.2	5
BHT	22.8 ± 4.2	85.2 ± 5.5	50
EEP	119.6 ± 20.5	86.5 ± 2.8	500

Values are means ± SEM (*n* = 2).
