# Supplementary material for: Antimicrobial, Antioxidant, Anti-Inflammatory, and Cytotoxic Activities of Propolis from the Stingless Bee Tetragonisca fiebrigi (Jataí)
Source: Evid Based Complement Alternat Med. 2015 Jun 22;2015:296186. doi: 10.1155/2015/296186 (PMC4491730; doi:10.1155/2015/296186)
Supplement: Supplementary file 1 — Graphical abstract: Presentation of the results more important of propolis from the T. fiebrigi. [file 296186.f1.pdf]

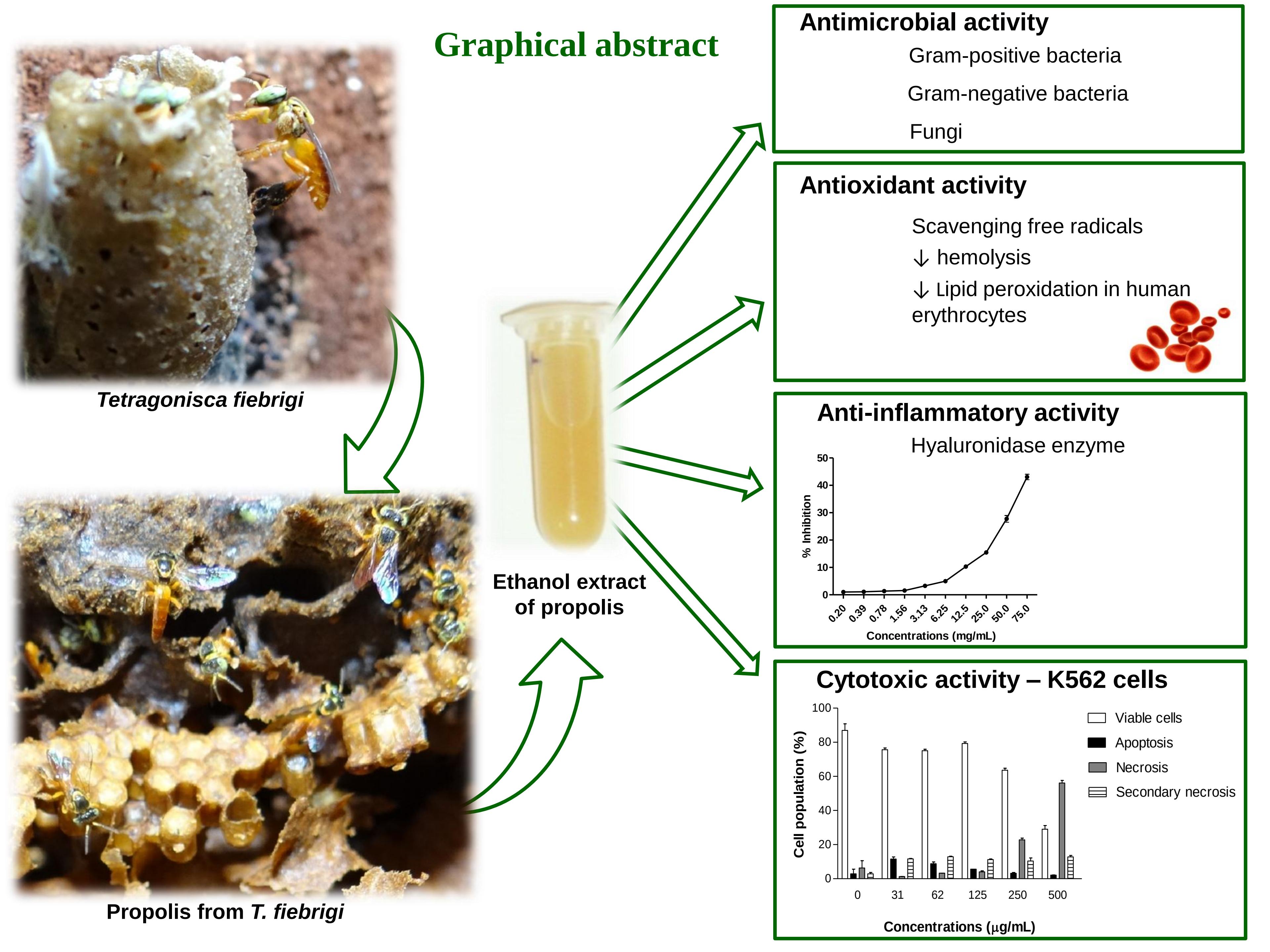
Graphical abstract
Tetragonisca fiebrigi
Propolis from T. fiebrigi
Ethanol extract of propolis
Antimicrobial activity
Gram-positive bacteria
Gram-negative bacteria
Fungi
Antioxidant activity
Scavenging free radicals
↓ hemolysis
↓ Lipid peroxidation in human erythrocytes
Anti-inflammatory activity
Hyaluronidase enzyme
% Inhibition
50
40
30
20
10
0
0.20
0.39
0.78
1.56
3.13
6.25
12.5
25.0
50.0
75.0
Concentrations (mg/mL)
Cytotoxic activity – K562 cells
Cell population (%)
100
80
60
40
20
0
31
62
125
250
500
Concentrations (µg/mL)
Viable cells
Apoptosis
Necrosis
Secondary necrosis
